# Highly Efficient Non-Enzymatic Glucose Sensor Based on CuO Modified Vertically-Grown ZnO Nanorods on Electrode

**DOI:** 10.1038/s41598-017-06064-8

**Published:** 2017-07-18

**Authors:** Rafiq Ahmad, Nirmalya Tripathy, Min-Sang Ahn, Kiesar Sideeq Bhat, Tahmineh Mahmoudi, Yousheng Wang, Jin-Young Yoo, Dae-Wook Kwon, Hwa-Young Yang, Yoon-Bong Hahn

**Affiliations:** 1 School of Semiconductor and Chemical Engineering, Nanomaterials Processing Research Center, Chonbuk National University, 567 Baekjedaero, Deokjin-gu, Jeonju-si, Jeollabuk-do 54896 Republic of Korea; 2 Department of BIN Fusion Technology, Chonbuk National University, 567 Baekjedaero, Deokjin-gu, Jeonju-si, Jeollabuk-do 54896 Republic of Korea

## Abstract

There is a major challenge to attach nanostructures on to the electrode surface while retaining their engineered morphology, high surface area, physiochemical features for promising sensing applications. In this study, we have grown vertically-aligned ZnO nanorods (NRs) on fluorine doped tin oxide (FTO) electrodes and decorated with CuO to achieve high-performance non-enzymatic glucose sensor. This unique CuO-ZnO NRs hybrid provides large surface area and an easy substrate penetrable structure facilitating enhanced electrochemical features towards glucose oxidation. As a result, fabricated electrodes exhibit high sensitivity (2961.7 μA mM^−1^ cm^−2^), linear range up to 8.45 mM, low limit of detection (0.40 μM), and short response time (<2 s), along with excellent reproducibility, repeatability, stability, selectivity, and applicability for glucose detection in human serum samples. Circumventing, the outstanding performance originating from CuO modified ZnO NRs acts as an efficient electrocatalyst for glucose detection and as well, provides new prospects to biomolecules detecting device fabrication.

## Introduction

In the current scenario, diabetes (resulting from insulin deficiency and characterized by abnormal blood glucose levels) is found to be greatly contributing to various leading causes of deaths worldwide^1, 2^. Thus in order to diagnose, maintain or prevent the life-threatening impact of diabetes, regular monitoring of blood glucose levels is always emphasized as a means of disease assessment and management. In the last few years, most researchers focused on the development of enzyme based electrochemical sensors, especially for glucose detection^3–8^. However, few shortcomings yet need to be overcome including complicated enzyme purification procedure and its high fabrication cost, lack of long-term stability due to enzyme denaturation, and low sensitivity owing to indirect electron transfer^9–11^. Addressing such formidable challenges, non-enzymatic electrochemical sensors have attracted significant interests for sensing various biomolecules to capitalize their direct electrocatalytic detection style and endorse cost-effective fabrication, high stability, and repeatability^12–15^. Of the various factors previously highlighted for establishing a high-performance non-enzymatic glucose sensor, an exquisite material choice and nanostructure optimization holds an effective strategy, since it enables exploitation of large surface area, high electrocatalytic activity, and effective electron transfer from electrocatalyst to conductive electrode substrate^16^. Recently, hybrid nanostructures (combination of two or more desired nanostructures) showed impressive characteristics with enhanced functions and improved performance in various applications. This motivated us to develop a hybrid nanostructure system employing a combination of different nanomaterials/nanostructures complementing each other in terms of desired specific features for high performance glucose sensor.

As a cost-effective nanomaterial with negligible toxicity, ZnO have shown great advantages for both enzymatic and non-enzymatic sensor application for biomolecules detection^17–19^. Especially for glucose sensing, ZnO nanostructures are universal choice of researchers because of their simple and easy synthesis at low temperature in different morphology with high crystallinity, good optical properties, excellent electrical characterstics^20, 21^. In our previous studies, we have shown that vertically-grown ZnO nanostructures on electrode surface have immense capability to hold enzymes because of their nanostructure morphology and high surface area, thereby enhancing overall sensing performance of the enzymatic sensors^22–24^. As well, we have also shown that these ZnO nanostructures can provide large surface area for nanostructure modification for the efficient non-enzymatic sensor devices^25^. Interestingly, a few studies on ZnO based hybrid nanostructures have shown enhanced catalytic activity owing to better surface-to-volume ratio of hybrid materials and fast electron transfer ability of ZnO to the supporting electrodes^26–28^. On the other hand, CuO nanostructures have been well-studied as an efficient material for the fabrication of non-enzymatic glucose detection owing to the fact that they possess excellent electrochemical and catalytic properties, inexpensive, low temperature, and easy tuning of CuO nanostructures, exhibiting potential outcomes on the sensors sensitivity due to their high surface and volume ratio^29–31^.

Keeping in view the various tedious steps in sensors fabrication, mostly previous protocols employed separately synthesized nanostructures that need binders to make slurry and coating for further utilization as an active electrode material for sensor fabrication. This approach further result in reduced electrocatalytic activity by blocking the catalytic active sites with binder, poor reproducibility and low stability of fabricated electrodes due to inhomogeneous and dense film of nanostructures produced via spin-coating/drop-casting. Therefore, fabrication of high performance sensing electrodes requires growth of nanostructures directly on the electrode surface that not only will seamlessly connect nanostructures with electrode but also promote fast electron transfer. To aid in the search for an easy approach to attach nanostructure on electrode surface, herein, we have directly grown ZnO NRs on FTO electrode surface (ZnO NRs/FTO) by low temperature hydrothermal method and functionalized with CuO (CuO-ZnO NRs/FTO electrode) to enhance the electrochemical activity for glucose detection via higher surface area and direct electron transfer. Morphological characterizations of CuO modified directly grown ZnO NRs (CuO-ZnO NRs) confirmed that the ZnO NRs are seamlessly connected to the electrode surface, vertically-oriented, and uniformly CuO-functionalized on the ZnO NRs. Further, the fabrication process was monitored with electrochemical impedance spectroscopy (EIS) measurements in order to get optimized CuO loading on ZnO NRs surface for delivering excellent catalytic properties during glucose detection. The electrochemical analysis of CuO-ZnO NRs electrocatalyst showed excellent electrochemical performance for glucose detection with high reproducibility and repeatability, stability, and selectivity. Additionally, the non-enzymatic sensors were also assessed for glucose detection in serum, illustrating its promising sensing applications in near-real/real time samples.

## Results

### Synthesis and structural properties

**Figure 1:**
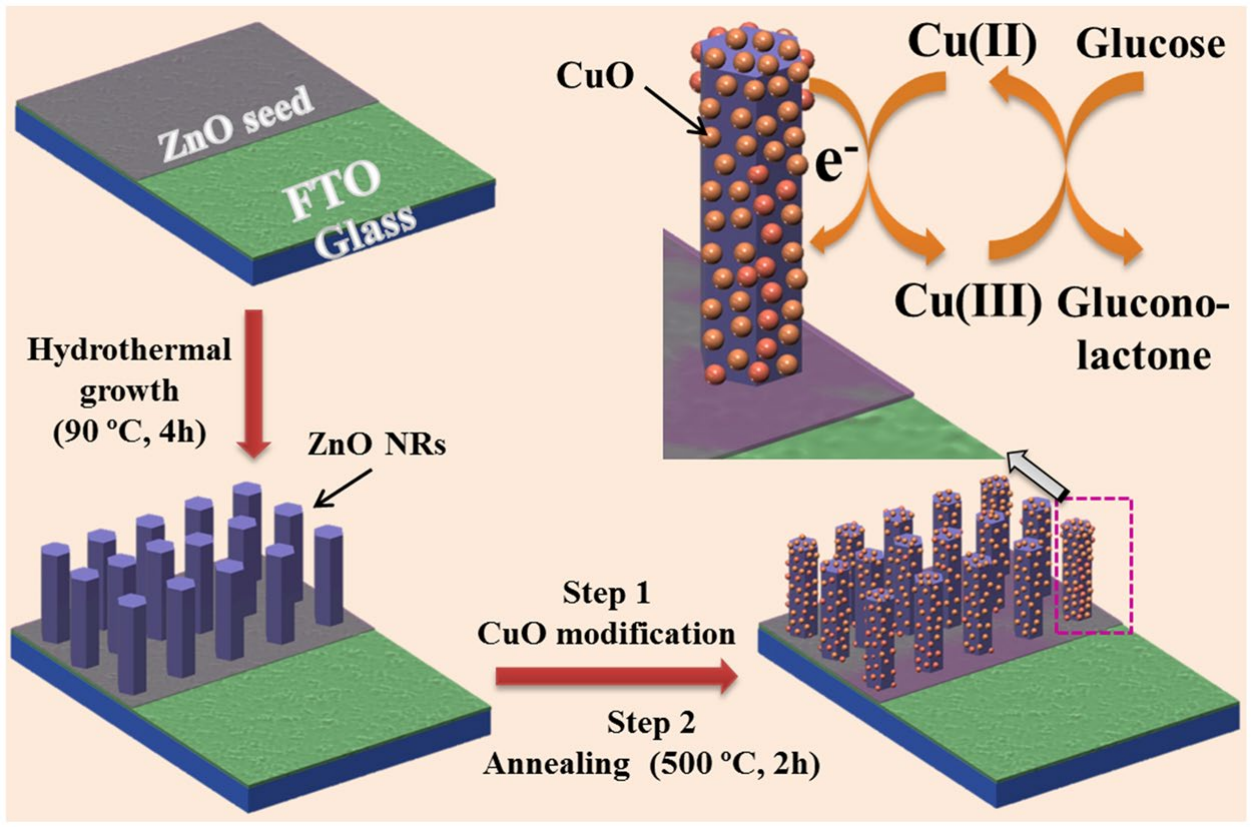
Schematic illustration. Non-enzymatic glucose sensor electrode fabrication and its application in glucose detection.

**Figure 2:**
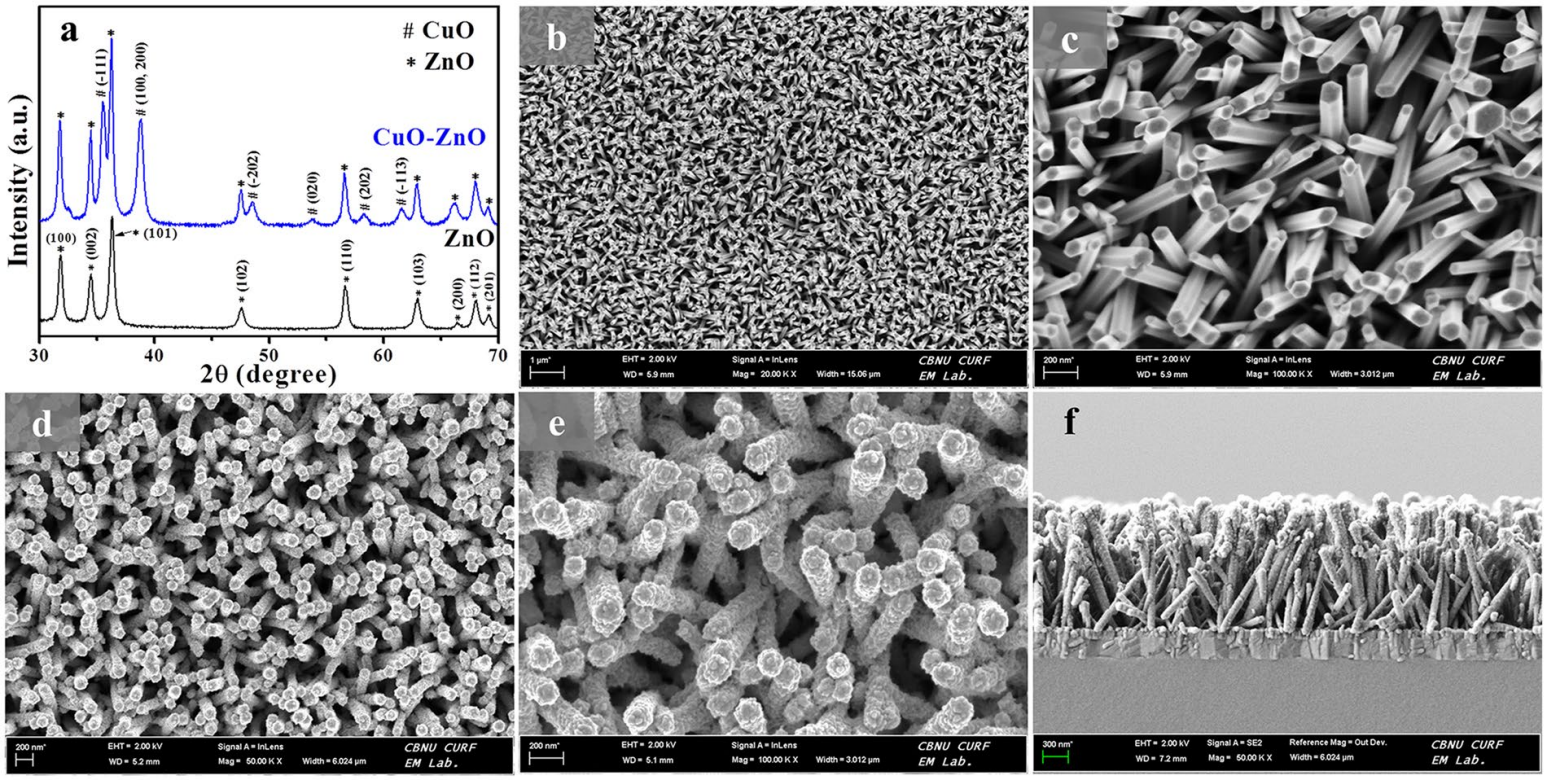
(**a**) XRD patterns of as-synthesized ZnO NRs and CuO modified ZnO NRs. (**b**) Low- and (**c**) high-resolution FESEM images of as-grown ZnO NRs. (**d**) Low-, (**e**) high-resolution, and (**f**) cross sectional FESEM view of CuO modified ZnO NRs.

Direct nanostructure synthesis and electrode fabrication strategy is briefly illustrated in Fig. 1 along with the details presented in method section. Hydrothermally grown ZnO NRs on electrode surface were modified with CuO through dip-coating and annealing. Figure 2a shows the XRD patterns of the as-synthesized ZnO NRs before and after CuO modification. For bare ZnO NRs spectrum, all diffraction peaks were well-indexed as hexagonal wurtzite structure of bulk ZnO (JCPDS 36–1451). After modification with CuO, the CuO-ZnO spectrum displays all the ZnO NRs diffraction peaks along the additional peaks corresponding to CuO modification and is indexed to the monoclinic CuO (JCPDS 48–1548). Next, morphologies of the as-synthesized ZnO NRs before and after CuO modification were examined by FESEM, as shown in Fig. 2b–f. From the images, the ZnO NRs were found to be uniformly and vertically grown on electrode surface in large scale. Compared to the smooth surface of bare ZnO NRs (Figure 2b–c), rough morphology was observed for CuO-ZnO NRs due to surface decoration with CuO (Fig. 2d–f). The cross-sectional image in Fig. 2f shows the length and diameter of ZnO NR as ~1.2 µm and ~80–90 nm, respectively.

Further observation of FESEM image was confirmed by TEM analysis (Supplementary Figure S1). TEM and their corresponding HRTEM images reveal that the smooth surface of ZnO NRs was uniformly and densely covered with CuO. The selected area electron diffraction (SAED) of ZnO (Supplementary Figure S1c) and CuO-ZnO NR (Supplementary Figure S1f) confirms the single-crystalline nature of ZnO and their preferential growth direction along the [0001] direction, the polar c-axis of the ZnO crystal lattice. From the SAED pattern of CuO-ZnO NR (Supplementary Figure S1f), additional diffraction rings were observed which coexist with the ZnO NR SAED pattern. These diffraction rings can be ascribed to the CuO modification over ZnO NR surface, confirming the well-formation of CuO-ZnO hybrid nanostructure. Further, CuO-ZnO hybrids were also analyzed with with TEM-EDX-line scan profile (Supplementary Figure S1g–j), which displays the corresponding elemental chemical composition and distribution as line scan profile, validating the presence of Cu (h, red line), Zn (i, green line), and O (j, blue line) elements in ZnO NR. The scan line positions of these elements demonstrates the presence of Cu (red line) and O (blue line) on the ZnO NR surface, mainly Cu (red line) intensity was higher at the edge of ZnO NRs indicating that the CuO was attached on to the ZnO NRs surface.

**Figure 3:**
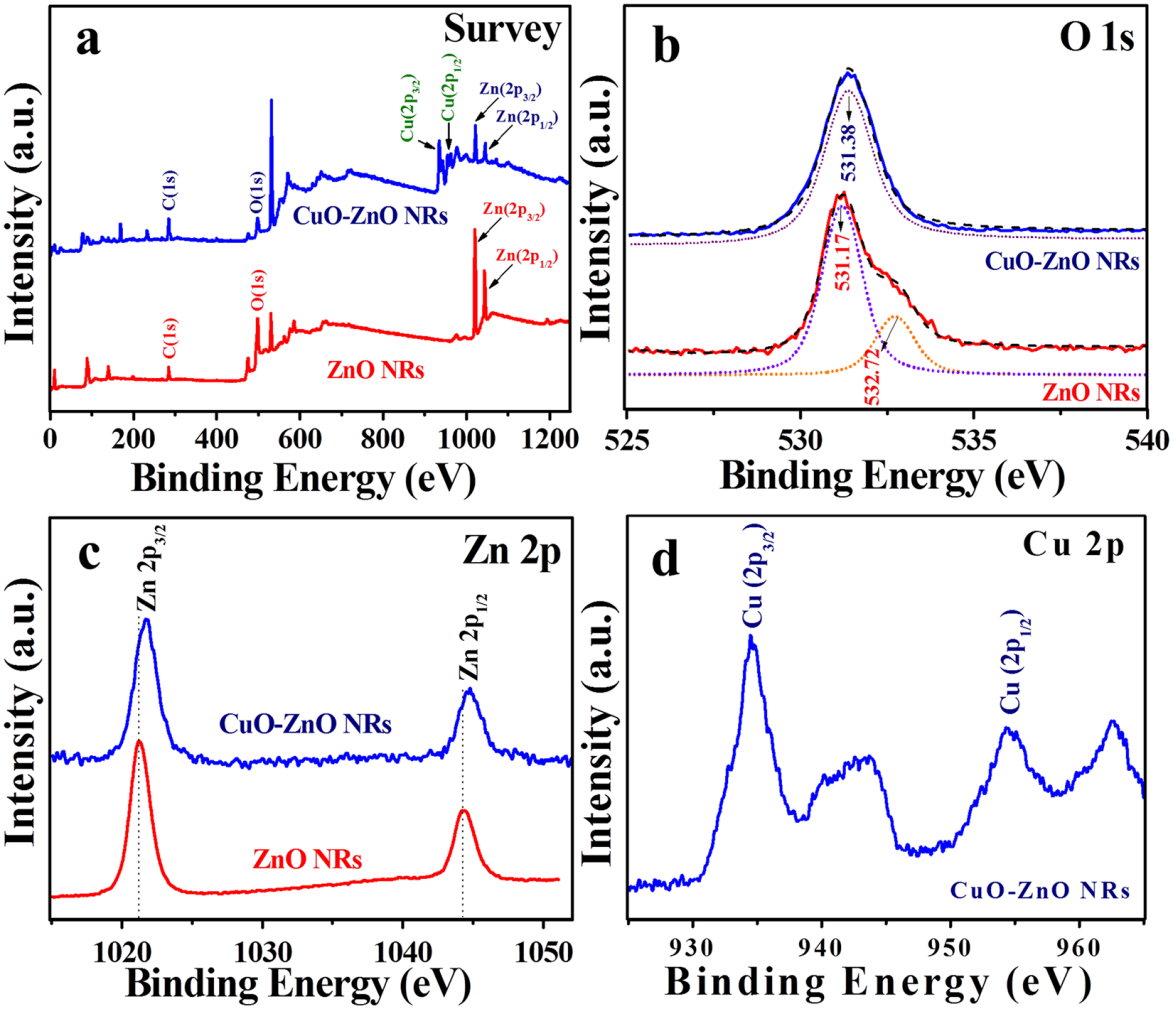
XPS analysis. XPS spectrum of ZnO NRs and CuO modified ZnO NRs showing full scan survey (**a**) and corresponding deconvoluted peaks in the high resolution spectra for O 1 s (**b**), Zn 2p (**c**), and Cu 2p (**d**) elements.

For characterizing detailed surface chemical compositions of ZnO NRs and CuO-ZnO NRs, XPS analysis was performed and shown in Fig. 3. The complete spectrum of ZnO NRs shows the presence of Zn, O, and C atoms only. However post-modification analysis shows that the CuO-ZnO NRs spectrum exhibits the presence of Cu, Zn, O, and C atoms, attributed to the successful modification of ZnO NRs with CuO (Fig. 3a). The corresponding high resolution spectra for O 1 s (Fig. 3b) displays two peaks for ZnO NRs sample at 531.17 eV and 532.72 eV, corresponding to the oxygen vacancies or defects (O_V_), presence of O^2^- species in the lattice (O_L_), and chemisorbed or dissociated (O_C_) oxygen species^32–34^. Compared to the pristine ZnO NRs, CuO-ZnO NRs showed a little shift to the higher binding energy which indicates the ZnO surface modification with CuO^12^. Further, both high resolution Zn 2p spectra of ZnO NRs and CuO modified ZnO NRs both showed two symmetric peaks (Fig. 3c). The ZnO NRs show peaks at around 1021.6 eV and around 1044.2 eV which corresponds to the Zn 2p_3/2_ and Zn 2p_1/2_, respectively. After CuO modification, both peaks of as-synthesized ZnO NRs were shifted to the higher binding energy, confirming the different chemical environment of as-synthesized CuO-ZnO NRs. In addition, to find out the presence of Cu^2+^ on ZnO NRs, the high resolution Cu 2p spectra of CuO-ZnO NRs were presented in the Fig. 3d. In the figure, there are two peaks located at approximately 934.6 eV and 954.3 eV, corresponds to the Cu 2p_3/2_ and Cu 2p_1/2_, respectively, that confirms the Cu^2+^ presence on ZnO NRs surface^35, 36^. Additionally, two shake-up satellite peaks for Cu 2p^3/2^ and Cu 2p^1/2^ were observed at higher binding energy at around 943.2 eV and 962.8 eV, attributed to the partially filled d-block (3d9) of Cu^2+^ and hence further confirms the formation of CuO over ZnO NRs surface^36^.

### Electrochemical properties

**Figure 4:**
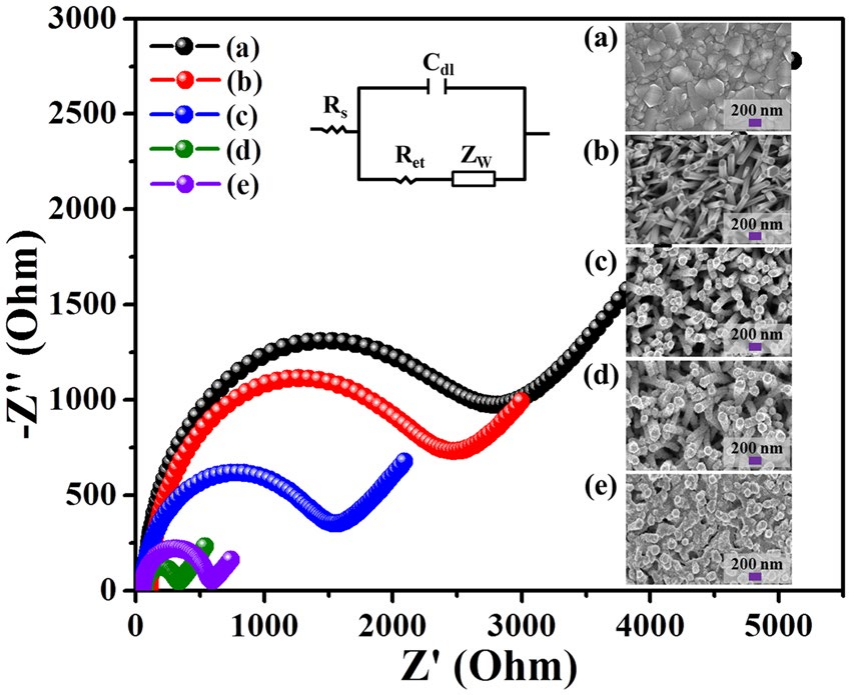
Typical Nyquist semicircle plots of EIS spectra. Electrode fabrication step measured in a mixture of 5 mM [Fe(CN)_6_]^3-/4-^ and 0.1 M KCl solutions at an applied amplitude of ± 5 mV within a frequency range of 0.01 Hz-100 MHz. (**a**) Bare FTO electrode, (**b**) ZnO NRs/FTO electrode, (**c**) 10 s CuO modified ZnO NRs/FTO electrode, (**d**) 20 s CuO modified ZnO NRs/FTO electrode, and (**e**) 30 s CuO modified ZnO NRs/FTO electrode. Inset FESEM images show the surface morphology of the electrodes. Randles equivalent circuit model is shown in inset, where C_dl_, R_s_, R_et_, and Z_w_ are double layer capacitor, solution resistor, electron transfer resistance, Warburg resistor, respectively.

Fig. 4 expresses the plotted impedance spectra of each electrode fabrication step along with the corresponding FESEM image in inset. The electrocatalytic behavior of each fabricated step was monitored by EIS measurements in a mixture of 5 mM [Fe(CN)_6_]^3-/4-^ and 0.1 M KCl solutions, and the impedance results were obtained using Randles equivalent circuit (see inset of Fig. 4). The bare FTO electrode (curve a) shows the largest Nyquist semicircle with the Ret of ~2250 Ω. However, after ZnO NRs growth on FTO electrode, the Nyquist semicircle becomes smaller and Ret value was decreased (R_et_ = ~2090 Ω), indicating improved electron transfer rate. In order to select the most efficient electrode, we modified ZnO NRs grown on FTO electrodes with CuO after dipping ZnO NRs/FTO electrode in precursor solution for 10, 20, and 30 s (curve c–e). After CuO modification, the CuO-ZnO NRs/FTO electrode showed much improved electron transfer rate. The best electrode with enhanced electron transfer rate (curve d, R_et_ = ~240 Ω) was obtained after dipping the ZnO NRs/FTO electrode in precursor solution for 20 s. Compared to 20 s treated CuO-ZnO NRs/FTO electrodes, 10 and 30 s modified CuO-ZnO NRs/FTO electrodes showed high R_et_ value of ~1320 and ~520 Ω, respectively. The result suggests that the controlled CuO-modification over ZnO NRs/FTO electrode is important, as less (curve c) and more (curve e) modification gives high electron transfer resistance. The low R_et_ reflects that the diffusion-limited process occurs between surface of electrode and the solution. Therefore, 20 s CuO-ZnO NRs/FTO electrodes were selected for further electrochemical characterizations and non-enzymatic glucose detection.

**Figure 5:**
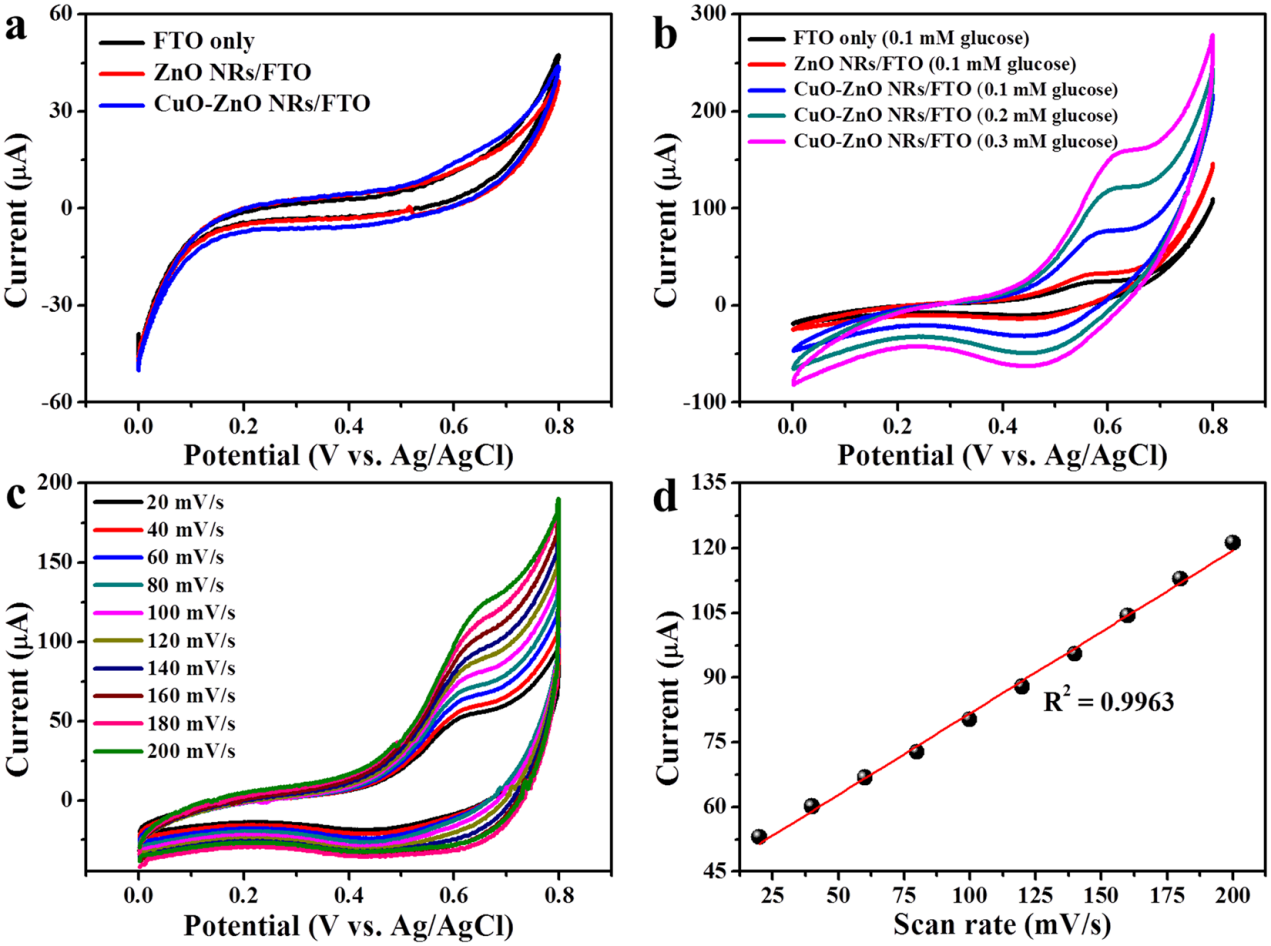
Typical CV curve of electrodes. (**a**) CV response of FTO, ZnO NRs/FTO, and CuO-ZnO NRs/FTO electrodes in blank NaOH solution (scan rat, 100 mVs^−1^). (**b**) CVs in the presence of glucose at different electrode (scan rat, 100 mVs^−1^). (**c**) CVs for CuO-ZnO NRs/FTO in 0.1 mM glucose at scan rats from 20 to 200 mVs^−1^. (**d**) Corresponding calibration plot of peak current *versus* scan rate.

The electrochemical tests for different electrodes (working electrodes) were performed in a three-electrode cell system with Pt wire as the counter electrode and Ag/AgCl as reference electrode within the potential range of 0 to +0.8 V. Fig. 5 presents the CV profile of electrochemical responses in 10 mL of 0.1 M NaOH solution with/without glucose. In the blank NaOH solution, no oxidation peak was observed (Fig. 5a). The addition of 0.1 mM glucose causes a negligible oxidation current for FTO and ZnO NRs/FTO electrodes (Fig. 5b) which confirms that these electrodes have no specific catalytic property like CuO modified electrodes. In contrast, CuO-ZnO NRs/FTO electrodes showed well-defined oxidation peak at +0.62 V in the presence of glucose. The obtained result clearly suggests that the oxidation peak corresponds to the electro-oxidation of glucose at the CuO modified electrode. The possible mechanism of non-enzymatic glucose detection on CuO modified electrode is presented in Fig. 1. The electrocatalytic oxidation of glucose can be ascribed to the conversion of Cu(II) to Cu(III) in NaOH solution, as suggested by Marioli and Kuwana^37^. In brief, during electrocatalytic oxidation of glucose, Cu(II) is electrochemically oxidized to Cu(III) which acts as an electron delivery system, and the glucose oxidized to gluconolactone is further oxidized to gluconic acid. In addition, CV response of CuO-ZnO NRs/FTO electrode was measured in 0.1 M NaOH solution at different scan rates from 20 to 200 mV s^−1^ in the presence of 0.1 mM glucose, as depicted in Fig. 5c. The corresponding calibration plot of peak current versus scan rate presented in Fig. 5d showed a linear change in current response with an increasing scan rate which indicates the surface-controlled electrochemical process over CuO-ZnO NRs/FTO electrode^38^.

### Analytical performance of non-enzymatic glucose sensor

**Figure 6:**
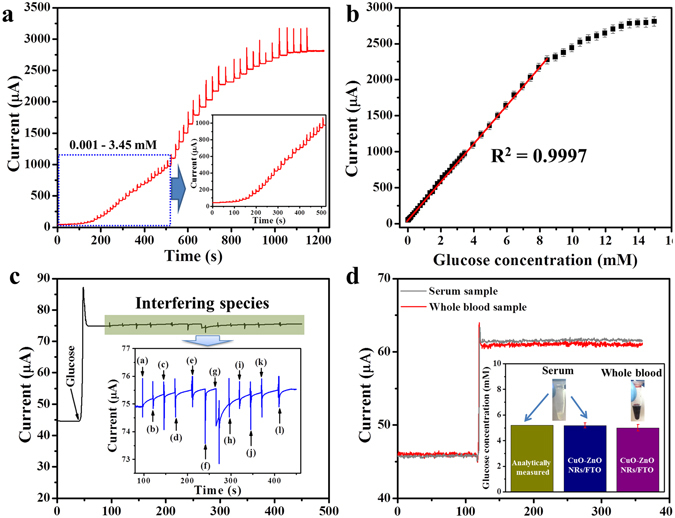
Non-enzymatic detection of glucose. (**a**) Amperometric response of CuO-ZnO NRs/FTO electrode at +0.62 V (versus Ag/AgCl) in 0.1 M NaOH solution with different glucose concentration from 0.001 to 14.95 mM and inset response curve shows the magnified view of low concentration range of glucose (0.001–3.95 mM). (**b**) Corresponding calibration plot of current response versus glucose concentration. (**c**) Anti-interference ability test. Amperometric response of the CuO-ZnO NRs/FTO electrode with the addition of 0.1 mM glucose and 0.02 Mm of each possible interfering species i.e. (a) AA, (b) UA, (c) DA, (d) NADH, (e) Mg^2+^, (f) Ca^2+^, (g) Cys, (h) NaCl, (i) lactose, (j) sucrose, (k) maltose, and (l) mannose in the 0.1 M NaOH solution at +0.62 V (*versus* Ag/AgCl). (**d**) Real sample glucose detection. Amperometric response of CuO-ZnO NRs/FTO electrode at +0.62 V (*versus* Ag/AgCl) in 9.5 mL 0.1 M NaOH solution after injecting 0.5 mL blood serum (i) and freshly drawn whole human blood (ii). The upper inset shows the photograph of serum and whole blood samples and lower inset shows the histogram of glucose concentration compared with blood chemistry analyzer result.

In order to demonstrate the analytical parameters (i.e. sensitivity, linear range, detection limit and response time), amperometric response of CuO-ZnO NRs/FTO electrode was performed at a fixed voltage of +0.62 V (*versus* Ag/AgCl) in 0.1 M NaOH solution by stepwise addition of glucose at different concentration. From the Fig. 6a, a well-defined and fast amperometric response for CuO-ZnO NRs/FTO electrode was noticed. Inset of Fig. 6a shows the current response at lower glucose concentration (0.001–3.45 mM). Upon addition of glucose, the current response quickly reached a steady-state and attains ~ 96% of response within 2 s. The response current was lineally increased with increasing glucose concentration, as shown in the calibration plot (current response *verses* glucose concentration) of amperometric response in Fig. 6b. The calibration plot was found to be linear in the concentration range of 0.001 to 8.45 mM with a correlation coefficient (R^2^) of 0.9997. Further, sensitivity was calculated as 2961.7 μA mM^−1^ cm^−2^ by dividing the slope of the linear portion of calibration curve with electrode surface area. The lower detection limit (LOD) was observed to be as low as 0.40 μM at the signal-to-noise ratio (S/N) of 3. A comparative analytical performance of our sensing electrode with previously published non-enzymatic glucose sensor is also presented in Table 1. As shown in Table, CuO-ZnO NRs/FTO electrode showed high sensitivity (2961.7 μA mM^−1^ cm^−2^) in wide linear range (0.001 to 8.45 mM), which is superior to previously reported values for CuO based sensor electrodes^12, 13, 16, 39–42^. Other parameters like response time and detection limits were also satisfactory. While comparing only sensitivity, our fabricated sensor showed less response than sensors fabricated using electrospinning and electrochemical anodization methods^12, 39, 42^. It is worthwhile to notice that the linear range of those sensors were ~4–16 folds less than our sensing electrodes. Overall, the enhanced sensing performance of non-enzymatic glucose sensor is ascribed to the direct growth of ZnO NRs on FTO electrodes which offers high surface area for CuO modification, resulting in fast electron transfer during electrochemical process of glucose oxidation occurring between the electrolyte and electrode. Importantly, we have used hydrothermal approach to fabricate non-enzymatic glucose sensing electrodes which bestow controllable nanostructures with good reproducibility and cost-effective fabrication process for stable glucose sensing devices.

**Table 1:** Comparison of the non-enzymatic electrochemical sensors based on direct modification of sensing electrodes.

Electrode	Methods	Sensitivity (µA cm^−2^ mM^−1^)	Linear range (mM)	Response time (s)	Detection limit (µM)	Ref.
CuO-ZnO NRs/FTO	Solution process	2961.8	0.001–8.45	<2	0.40	This work
Porous ZnO-CuO hierarchical nanocomposites/FTO	Electrospinning	3066.4	0.00047–1.60	1.2	0.21	12
CuO nanoparticles/Ag	Inkjet printing	2762.5	0.05–18.45	<2	~0.5	13
CuO nanowires/Cu foam	Electrochemical anodization	2217.4	0.001–18.80	—	0.30	16
CuO-NiO microfibers/FTO	Electrospinning	3165.53	0.003–0.51	<3	0.001	39
Cu nanocubes/MWCTs/GCE	Electrodeposition	1096	Up to 7.50	~2	1.0	40
Ag/CuO NFs-ITO	Electrospinning	1347	0.0005–0.55	<3	0.0517	41
CuO Nanothorns/CuO/Cu foam	Electrochemical anodization	5984.26	0.0002–2	<5	0.275	42

### Anti-interference ability, reproducibility, reusability, and stability tests

Anti-interference ability of non-enzymatic based glucose sensing devices is a major challenge, which could affect the electrode’s sensing performance. Herein, to verify the selectivity of CuO-ZnO NRs/FTO electrode in the presence of interfering species (such as AA, UA, DA, NADH, Mg^2+^, Ca^2+^, Cys, NaCl, lactose, sucrose, maltose, and mannose), the amperometric response of the sensing electrode was recorded with the addition of 0.1 mM glucose and 0.02 mM of each above interfering species in the 0.1 M NaOH solution at +0.62 V (*versus* Ag/AgCl), shown in Fig. 6c. The addition of 0.1 mM glucose resulted in a rapid current response, whereas interfering species addition showed negligible current responses. A magnified view of each interfering species addition and current response is shown in the inset of Fig. 6c, which confirms the negligible current responses compared to 0.1 mM glucose. These results suggest the suitability of CuO-ZnO NRs/FTO electrode for the selective detection of glucose in real blood samples.

Reproducibility, reusability, and stability are other vital parameters for measuring the efficiency of sensing devices. The reproducibility of CuO-ZnO NRs/FTO electrode was investigated by employing 10 freshly prepared non-enzymatic glucose sensors. Their CV response was recorded in 0.1 M NaOH solution containing 0.1 mM glucose at the scan rate of 100 mVs^−1^ (Supplementary Figure S2a). The peak current of CV response is presented in Supplementary Figure S2b, which showed relative standard deviation (RSD) of 4.6%. Similarly, the reusability CuO-ZnO NRs/FTO electrode was measured in 10 samples, each containing 0.1 mM of glucose at 100 mVs^−1^ scan rate and the obtained data is shown in Supplementary Figure S2c and S2b. After 10 times usage, CuO-ZnO NRs/FTO glucose sensing electrode retains around 98% of its original response, suggesting excellent reproducibility and reusability of our sensing electrode. In addition, the long-term stability of the CuO-ZnO NRs/FTO electrode was explored by measuring CV response in the presence of 0.1 mM glucose in 0.1 M NaOH solution at the scan rate of 100 mV s^−1^ (Supplementary Figure S2e). The CV response was evaluated once every three days and after completion of measurement, the electrodes were stored at room temperature. As shown in the histogram (Supplementary Figure S2f), after 30 days of usage the current response was found to be 93.2% of its original response (measured after fabricating the electrode). The excellent stability of electrode was due to the directly grown nanorods on electrode surface, which provided robust mechanical stability to the sensing device.

### Non-enzymatic glucose detection in real human blood

The excellent sensing performance of CuO-ZnO NRs/FTO electrode suggests the suitability for glucose detection in real samples for future practical applications. In order to justify the above statement, we measured amperometric response of CuO-ZnO NRs/FTO electrode in 9.5 mL of 0.1 M NaOH solution after injecting 0.5 mL blood serum and freshly drawn whole human blood at an applied potential of +0.62 V (*versus* Ag/AgCl), shown in Fig. 6d. The blood samples used in the experiments were obtained from same donor, which was also analyzed using blood chemistry analyzer VetScan VS2 (Abaxis, Inc., Union City, CA 94587) and the obtained data (5.2 mM glucose concentration) was further compared (inset of Fig. 6d). As presented in histogram, the measured glucose concentration in the serum sample was in good agreement with the analytically measured value. However, whole human blood sample showed ~4% less concentration of glucose due to the presence of different types of molecules (such as cells, protein fragments, etc.)^43^. Therefore, as-prepared CuO-ZnO NRs/FTO electrode may hold potential practical application for glucose detection in real samples.

## Discussion

We have successfully presented the fabrication of highly-efficient non-enzymatic glucose sensor electrode by directly growing ZnO NRs on FTO electrode followed by CuO modification. The uniqueness of CuO-ZnO NRs/FTO electrode is that, the directly-grown ZnO NRs on electrode surface provides easy substrate penetrable structure, and large surface area for CuO modification which in turn enhances electrochemical activity for glucose detection. The sensing electrode exhibited remarkable high performance in terms of sensitivity, wide response range, response time, selectivity, reproducibility, repeatability, and stability. Additionally, the glucose detection in real human blood shows the electrodes suitability for practical or real-time applications. This improved sensing performance is mainly attributed to the directly grown nanostructures that provide an excellent contact between the nanostructure and electrode with high surface area for catalytic sites, facilitating suitable path for electron transport during electrochemical activity^16, 40, 42, 44–55^. Overall, the fabricated electrodes can be envisioned as promising design for practical application of non-enzymatic glucose measurement in real clinical samples which may garner considerable benefits for different biomolecule detection.

## Methods

### Reagents

Fluorine doped tin oxide (FTO) coated glass electrode, Zinc nitrate hexahydrate (Zn(NO_3_)_2_•6H_2_O, 98%), hexamethylenetetramine (HMTA; C_6_H_12_N_4_, ≥99.0%), copper(II) sulfate pentahydrate (CuSO_4_•5H_2_O, ≥98.0%), Nafion (5 wt.% in lower aliphatic alcohol and water mixture), sodium hydroxide (NaOH, ≥97.0%), D-(+)-Glucose (C_6_H_12_O_6_, 99.5%), ascorbic acid (AA), uric acid (UA), dopamine (DA), β-Nicotinamide adenine dinucleotide (NADH), magnesium chloride (MgCl_2_, ≥98%), calcium chloride (CaCl_2_) solution, L-cysteine (L-cys), sodium chloride (NaCl, ≥99.5%), lactose, sucrose, maltose, and mannose were purchased from Sigma-Aldrich. All the chemicals used in this study were of analytical reagent and used as received without further purification. All solutions were prepared with ultra-pure water (18 MΩ cm), obtained from a micro pure HIQ water purifying system.

### Fabrication of non-enzymatic glucose sensor electrodes

To fabricate non-enzymatic glucose sensor electrodes, first, FTO (3 × 0.3 cm) glasses were cleaned using detergent, water, acetone, ethanol, and dried. Before growing ZnO NRs, a thin layer (~50 nm) of ZnO seed was deposited on the selected area (0.4 × 0.3 cm, rest of the substrate area was covered with tape) of FTO using ZnO as a sputtering target. Then, ZnO NRs were grown on seeded FTO via hydrothermal method after suspending substrates upside down in a Pyrex glass bottle at 90 °C for 4 h containing 50 mL deionized water with an equal molar solutions of Zn(NO_3_)_2_•6H_2_O (0.05 M) and HMTA (0.05 M). After completion of reaction time, ZnO NRs grown on FTO electrodes were rinsed to remove impurities prior to modification. In the next step, the ZnO NRs grown on FTO electrodes were modified; a precursor solution of CuSO_4_•5H_2_O (1.8 gm) was prepared in 25 mL deionized water and ZnO NRs grown on FTO electrodes were put in the prepared solution for 10, 20, and 30 s, respectively. Then the electrodes were air-dried and finally CuO nanoparticles (NPs) modified ZnO NRs grown on FTO electrodes were annealed at 500 °C for 2 h. Before electrochemical measurements, the fabricated electrodes surfaces were covered with 2 μL of 0.5 wt% Nafion solution to eliminate the possible fouling and reduce the interferences effect.

### Characterization and measurements

The surface morphology of the as-synthesized ZnO NRs before and after CuO modification was inspected using field emission scanning electron microscopy (FESEM, Hitachi S4700, and SUPRA 40VP), transmission electron microscopy (TEM), and high-resolution transmission electron microscopy (HRTEM) equipped with digital charge-coupled device (JEOL-JEM-2010 equipped with CCD camera). The elemental chemical composition was determined by TEM-EDX-line scan. The crystalline structure of ZnO NRs before and after CuO modification was analyzed using X-ray diffractometer (XRD, Rigaku) with Cu-Kα radiation (λ = 1.54178 Ǻ) in the range of 30–70° with 8°/min scanning speed. The chemical states were analyzed by X-ray photoelectron spectroscopy (M/s. AXIS-NOVA, Kratos Inc.) using X-ray source of monochromatic Al K (1486.6 eV) 150 W. All the electrochemical measurements were conducted at room temperature using an electrochemical measurement station (Ivium CompactStat.e; Ivium Technologies) connected to computer with a conventional three-electrode cell system: a working electrode (Nafion/CuO-ZnO NRs/FTO), platinum (Pt) wire as counter electrode, and Ag/AgCl as reference electrode. During amperometric measurements, solution was continuously stirred at 150 rpm. The electrochemical impedance spectroscopy (EIS) measurements were performed in a mixture of 5 mM [Fe(CN)_6_]^3-/4-^ and 0.1 M KCl solutions. The EIS measurements were taken within a frequency range of 0.01 Hz–100 MHz with applied amplitude of ± 5 mV.

## Electronic supplementary material

Supplementary information 
