# Supplementary material for: Highly Efficient Non-Enzymatic Glucose Sensor Based on CuO Modified Vertically-Grown ZnO Nanorods on Electrode
Source: Sci Rep. 2017 Jul 18;7:5715. doi: 10.1038/s41598-017-06064-8 (PMC5515932; doi:10.1038/s41598-017-06064-8)
Supplement: Supplementary file 1 — Supplementary information [file 41598_2017_6064_MOESM1_ESM.pdf]

Supplementary information

Highly Efficient Non-Enzymatic Glucose Sensor Based on CuO Modified Vertically-Grown ZnO Nanorods on Electrode

Rafiq Ahmad[1], Nirmalya Tripathy[2], Min-Sang Ahn[1], Kiesar Sideeq Bhat[1], Tahmineh Mahmoudi[1], Yousheng Wang[1], Jin-Young Yoo[1], Dae-Wook Kwon[1], Hwa-Young Yang[1] & Yoon-Bong Hahn[1,*]

[1]School of Semiconductor and Chemical Engineering, Nanomaterials Processing Research Center, Chonbuk National University, 567 Baekjedaero, Deokjin-gu, Jeonju-si, Jeollabuk-do, 54896, Republic of Korea

[2]Department of BIN Fusion Technology, Chonbuk National University, 567 Baekjedaero, Deokjin-gu, Jeonju-si, Jeollabuk-do, 54896, Republic of Korea.

* **Corresponding author**. Tel.: +82 63 2702439; Fax: +82-63-270-2360.

E-mail: ybhahn@chonbuk.ac.kr (Y.-B. Hahn)

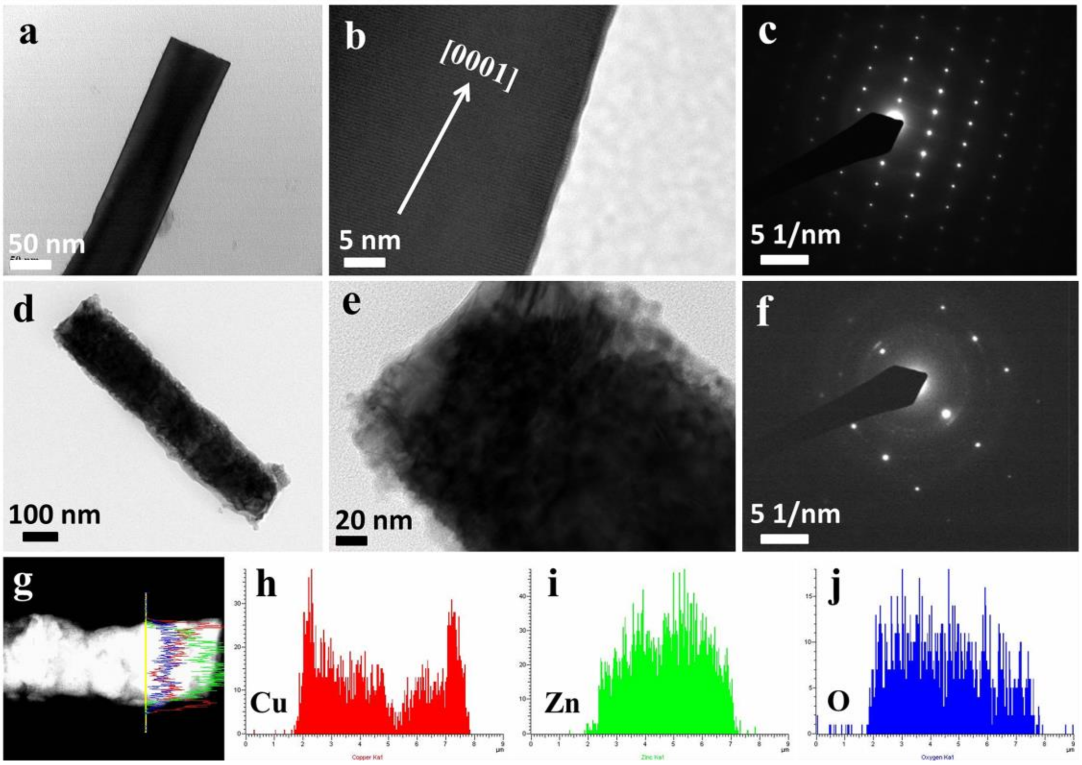

Supplementary Figure S1. TEM images (a and d) and HRTEM images (b and e) of ZnO NR and CuO modified ZnO NR, respectively. SAED pattern of as-synthesized ZnO NR (c) and CuO modified ZnO NR (f), and elemental mapping image of CuO modified ZnO NR (g) showing TEM-EDX-line scan profile of Cu (h), Zn (i) and O (j), respectively.

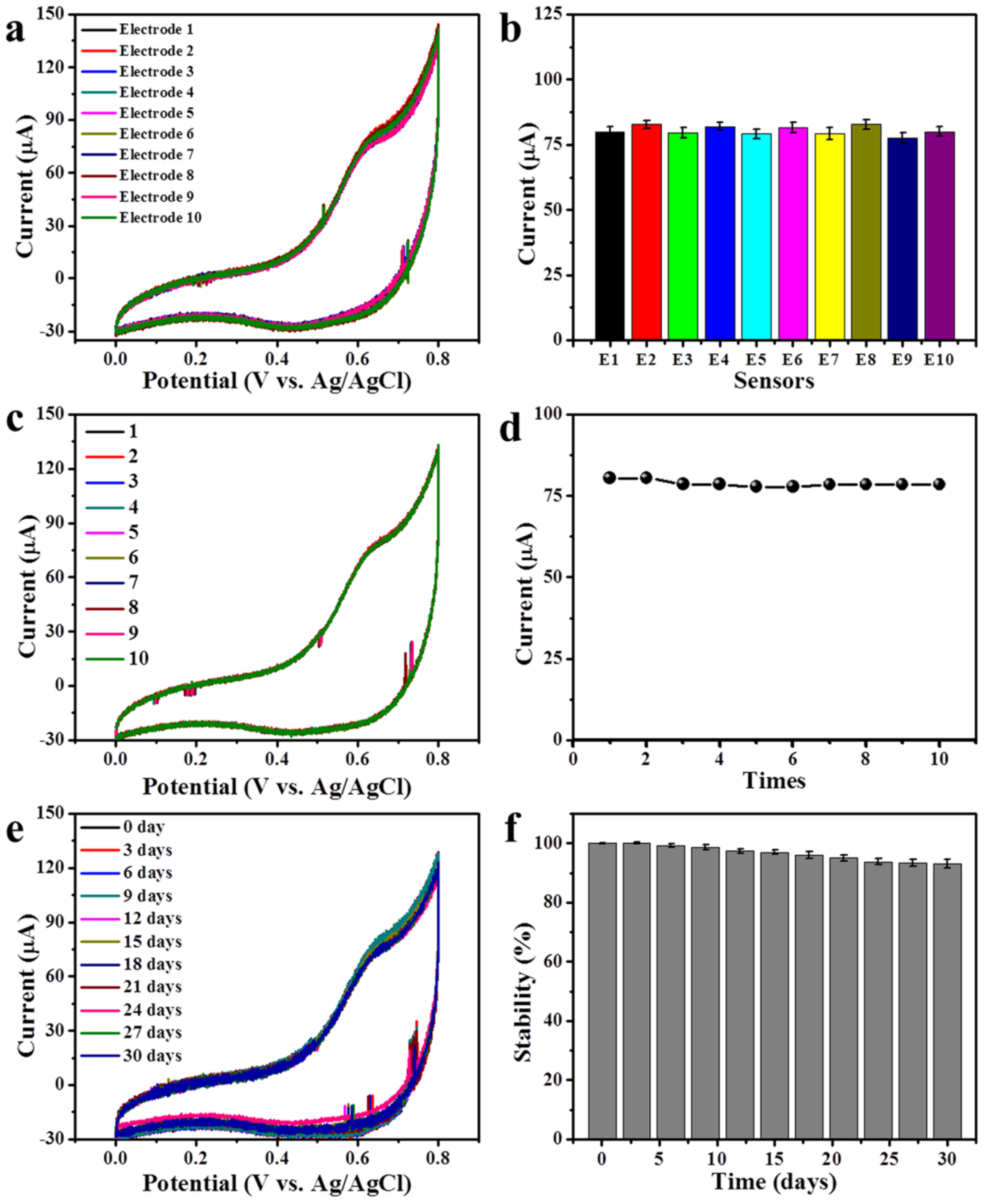

Supplementary Figure S2. Reproducibility (a-b), reusability (c-d), and stability (e-f) tests. All the tests were performed using CuO-ZnO NRs/FTO electrodes in 0.1 M NaOH solution containing 0.1 mM glucose at the scan rate of 100 mV s^{-1}.
